# A Low-Cost IoT-Based System to Monitor the Location of a Whole Herd

**DOI:** 10.3390/s19102298

**Published:** 2019-05-18

**Authors:** Francisco Maroto-Molina, Jorge Navarro-García, Karen Príncipe-Aguirre, Ignacio Gómez-Maqueda, José E. Guerrero-Ginel, Ana Garrido-Varo, Dolores C. Pérez-Marín

**Affiliations:** 1 Department of Animal Production, School of Agricultural and Forestry Engineering (ETSIAM), University of Cordoba, 14014 Cordoba, Spain; pa1gugij@uco.es (J.E.G.-G.); pa1gavaa@uco.es (A.G.-V.); dcperez@uco.es (D.C.P.-M.); 2 Data Science Laboratory, School of Computer Engineering, University Rey Juan Carlos, 28933 Mostoles, Spain; j.navarro.2016@alumnos.urjc.es (J.N.-G.); k.principe@alumnos.urjc.es (K.P.-A.); 3 SensoWave SL, 28830 San Fernando de Henares, Spain; imaqueda@sensowave.com

**Keywords:** precision livestock farming, global positioning system, bluetooth low energy, low-power wide-area network, wireless sensor network

## Abstract

Animal location technologies have evolved considerably in the last 60 years. Nowadays, animal tracking solutions based on global positioning systems (GPS) are commercially available. However, existing devices have several constraints, mostly related to wireless data transmission and financial cost, which make impractical the monitorization of all the animals in a herd. The main objective of this work is to develop a low-cost solution to enable the monitorization of a whole herd. An IoT-based system, which requires some animals of the herd being fitted with GPS collars connected to a Sigfox network and the rest with low-cost Bluetooth tags, has been developed. Its performance has been tested in two commercial farms, raising sheep and beef cattle, through the monitorization of 50 females in each case. Several collar/tag ratios, which define the cost per animal of the solution, have been simulated. Results demonstrate that a low collar/tag ratio enable the monitorization of a whole sheep herd. A larger ratio is needed for beef cows because of their grazing behavior. Nevertheless, the optimal ratio depends on the purpose of location data. Large variability has been observed for the number of hourly and daily messages from collars and tags. The system effectiveness for the monitorization of all the animals in a herd has been certainly proved.

## 1. Introduction

Animal location and tracking have been topics of interest for both wildlife and livestock researchers for a long time. Animal location recording allows researchers to evaluate some key aspects, such as the movements of animals around the landscape [1], the spatial heterogeneity of field occupancy by animals [2], the pasture utilization, the animal performance and behavior [3], or the social affiliations within a herd [4,5].

Early methods for animal location relied on human observation of natural (color patterns) or artificial features (colored collar or tag). Problems of these methods included observer fatigue and associated error, physical limitations, external factors (weather and light), and the effect of observer proximity on animal behavior [3]. Since the late 1950s, very high frequency (VHF) tracking technologies have been commercially available to gather location data [6]. However, VHF-based tracking systems also have several limitations, from, e.g., disturbing the animal to only being able to track during the day because of the difficulty of the terrain. Location fixes and accuracy depend on the position of the researcher (ground or air), his discomfort and fatigue, his visibility, and the characteristics of the terrain. Without a visual sighting, errors may exceed 500 m [6].

Commercial development of telemetry systems based on global positioning systems (GPS) began in 1991, offering researchers the opportunity to overcome most of the limitations listed above. GPS-based systems have the potential to collect large amounts of high-quality location data 24 h per day and under all weather conditions. The accuracy of GPS tracking systems is generally considered to range from 5–30 m [7,8], depending on vegetation cover, topography and even animal position [9]. Thus, a new era of animal tracking began after the commercialization of GPS-based systems, and scientific literature contains numerous examples of successful studies using GPS positioning [10,11,12].

The first GPS animal units were fitted with a 400 g lithium battery pack that was expected to provide one year of operation at eight fix attempts per day, although field trials downgraded life expectancies to provide 268 days of service at six fixes per day [13]. The total weight of these first units was 1.8 kg, which limited their use to research studies on large mammals. Due to advances in the miniaturization of sensor technologies and to the development of higher energy-dense batteries, some devices enabling the tracking of smaller species, e.g., birds, are now available [14]. Nonetheless, even with current devices, the size of the battery pack is critical in finding a threshold between the temporal resolution and the operational life.

Another important drawback of most early GPS-based systems was that data were stored on board until the unit could be remotely released by a radio-activated “break-away” mechanism or recovered by the recapture of the animal. The development of mobile communications has enabled the transfer and regular updating of location data by GSM/SMS or GSM/GPRS services [15]. GSM services are widely available in Europe, but there are many vast areas throughout the world, mostly rural areas, without them. Opportunistic sensor networks, based on peer-to-peer routing, have been used to gather location data of several animals when GSM services were not available [16]. However, regular updating of the information can be challenging in that case.

The Internet of Things (IoT) is a paradigm shift in the enhancement of the connectivity of the Internet. It provides the technical feasibility for a large number of sensors, actuators, and smart devices to remain connected with it, which is key for the development of real-time or quasi-real-time applications. The requirements of IoT applications have driven the emergence of low power wide area (LPWA) networks. They provide long-range communication up to 10–40 km in rural areas and 1–5 km in urban areas. Thus, an entire city or several farms can be covered by a single base station [17]. In addition, LPWA networks are highly energy efficient and inexpensive, with a radio chipset cost less than 2 € and an operating cost of 1 € per device [18]. Hereafter, it is expected that the 5th generation (5G) of wireless mobile communications will provide the means to allow an all-connected world of humans and devices, which would lead to a global LPWAN solution for IoT applications [17].

Precision livestock farming (PLF) aims to manage individual animals by continuous and real-time monitoring of their health, welfare, production/reproduction, and/or environmental impact [19]. Despite the advances in GPS-based technologies for animal tracking in recent years, several constraints limit their use as PLF tools in commercial farms, especially in the case of extensive production systems. First, wireless data transmission is challenging in most rural areas. As described in the previous paragraph, IoT solutions are an opportunity to overcome that problem, but there are still few IoT developments devoted to animal tracking [20,21]. On the other hand, the financial cost of GPS tracking systems (generally several hundreds of euros per unit) remains a problem for widespread use of these technologies [22]. This is especially relevant in small ruminants, as their individual cost is low when compared to the tracking device cost. At best, some animals within the herd are monitored by GPS devices, which could be sufficient for some utilities, e.g., the monitorization of the percentage of time devoted to rest, graze, and travel [23,24]. Nevertheless, most authors argue that few animals are not representative of an entire herd [3,25,26]. Additionally, there are some utilities that would require knowing the location of all the animals in a herd, e.g., theft control, heat detection, or calving monitoring.

The main objective of this paper is to develop a low-cost solution that enables the monitoring of the location of all the animals in a herd and the continuous updating of location data, which is needed for farmer decision support. The secondary objective is to test the real-world performance of the solution in commercial farms with different production conditions.

The rest of the paper is organized as follows: Section 2 presents the hardware components of the solution and describes the methodology of system validation. Section 3 presents the results obtained regarding system functioning, configuration and validation in commercial farms. In Section 4, the results are discussed, and new utilities of the solution are proposed. Conclusions and further research needs are presented in Section 5.

## 2. Materials and Methods

### 2.1. System Components

For gathering, transmitting, and processing the location data of all animals in a herd, a cost-effective solution has been developed. The system is based on the integration of two devices: GPS collars connected to a LPWA network (Sigfox, Labège, France) and low-cost Bluetooth low energy (BLE) tags connected to those collars. The system requires some animals in the herd being fitted with GPS collars and the rest with BLE tags. The collar/tag ratio defines the cost per animal of the solution for tracking the location of the entire herd. Every GPS collar gathers data about the location of the animal fitted with such a device. At the same time, collars record the identification of the BLE tags situated around them, enabling the user to know the approximate location of animals equipped with BLE tags. Data about the collar location and identification of nearby tags are transmitted to a server using Sigfox, where they are stored and analyzed.

The GPS collar has the following hardware elements: a long-range IoT communications module based on Sigfox technology that allows the transmission of messages between the collar and the server; a short-range communications module based on BLE technology that enables the reading of the advertisement messages coming from the BLE tags (this module can record the identification of the BLE tag and the received power); a GPS unit to gather location data; a microcontroller with the low-level firmware running on it; a lithium battery pack; and an IP67 enclosure.

BLE tags consist of a Bluetooth 4.2 communications module, a microcontroller, a coin battery and an IP67 enclosure. The module is configured to send advertisement messages using channels 37, 38, and 39. The message sent by each BLE tag consists of an advertisement that includes the device identification and a code referring to the device owner. Thus, two BLE tags could have the same identification code if their owners were different.

The transmission of the information gathered by the solution to the cloud platform uses a MQTT connectivity protocol. The cloud platform is based on FIWARE components. Metadata are managed with Orion Context Broker through NGSI data models in order to ensure data interoperability. Farmers get location data through a multiplatform app and may configure some alerts, e.g., “animal outside the plot”.

**Figure 1:**
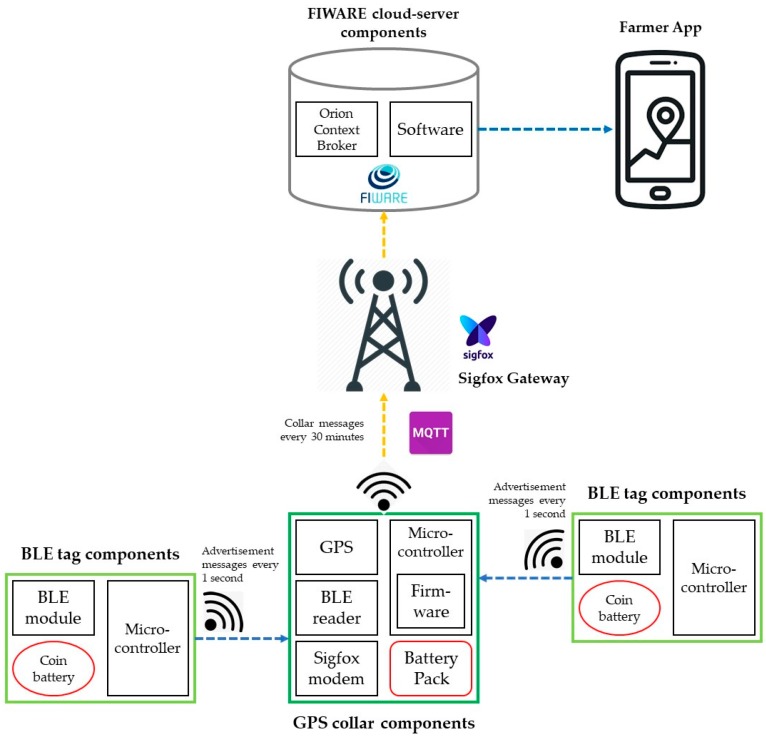
Architecture diagram of the proposed solution.

Figure 1 summarizes the main elements of the system architecture.

### 2.2. Testbeds

Two commercial farms have been used as testbeds to validate the performance of the proposed solution under different livestock production conditions.

Both testbeds are situated in Andalusia, Spain. Andalusia is a semi-arid Mediterranean region where rain is scarce and irregularly distributed over the year. Average temperature is 18 °C and annual precipitation ranges 400–600 mm. Herbs are the main forage resource, basically in fall and spring when rainfall occurs [27]. Moreover, both farms are located in “dehesas”, which are defined as agrosilvopastoral systems, originated from the clearing of holm oak and cork woodlands, mostly used for livestock farming [28].

The first farm was situated in Northern Cordoba (38.49° N, 5.11° W). The validation study was carried out with a “Merina” sheep herd composed of 50 ewes. From July to October 2018, for 100 consecutive days, 25 randomly selected ewes were fitted with GPS collars and another 25 with BLE tags. During the experiment, ewes grazed free-range across 70 hectares. The same experimental protocol was used in the second farm, located in Northern Huelva (37.96° N, 6.25° W), with a “Negra Andaluza” beef cattle herd. Twenty-five cows were fitted with GPS collars and another 25 with BLE tags. In this case, beef cows grazed free-range on 300 hectares from November 2018 to February 2019. Both “Merina” and “Negra Andaluza” are autochthonous breeds, highly adapted to the production conditions of “dehesa” farms.

### 2.3. Data Analysis

Different collar/tag ratios, from 1/25 to 25/25, have been simulated in order to study the changes in the following key performance indicators (KPIs) of the system: average number of BLE tag messages received per day, average number of BLE tags detected by all the GPS collars over a day, average time between two consecutive messages from the same BLE tag, average number of BLE tags included in each message sent by GPS collars, and the percentage of collar messages received without BLE tag information.

The average values of KPIs over the experiment have been calculated for each collar/tag ratio by randomly selecting the correspondent number of collars. The random selection process has been repeated 5000 times, following the recommendations of [29]. Both average and standard deviation have been calculated for each KPI. The same procedure has been used to study the evolution of KPIs over the experiment and within an average day.

For the graphics included in the present paper, a trend line was calculated and drawn to make charts more easily understandable. Generalized additive models [30] were used with a basis of 10 for daily charts and 15 for hourly ones.

R Project [31] has been used for data handling, calculations, and graphics.

## 3. Results

### 3.1. System Functioning

The system functioning can be described as follows: (1) the GPS unit is in an idle state (low power consumption) most of the time; (2) the GPS unit is activated and gathers location data every several minutes (depending on the configuration); (3) simultaneously, the BLE communications module, which has been set up to maximize the reading range (>75 m depending on habitat characteristics), scans the surrounding tags during several seconds (depending on the configuration); (4) the collar composes the information coming from the GPS unit and the BLE module to be sent through the Sigfox communications module; and (5) the messages sent by GPS collars are received by the server of the cloud solution, where they are extracted and analyzed. First, the encoded data from the collars are decoded to recompose the collar location data and the identification-power pairs of the tags. Once this information has been extracted, it is analyzed and stored in databases.

As mentioned in Section 2.1, GPS collars use Sigfox technology to send the data to the server. Sigfox is a LPWA network operator that uses binary phase-shift keying (BSPK) modulation in an ultra-narrow band (100 Hz) and unlicensed ISM bands, e.g., 868 MHz in Europe, 915 MHz in North America, and 433 MHz in Asia [17]. The use of Sigfox involves two challenges for the proposed solution. The first one is that the maximum size of each message is 12 bytes. The second one is the duty cycle of the transmission technology. In the case of the ISM band used by Sigfox, the limitation is a maximum use of 1% each hour, which translates into a maximum number of messages of 140 per day (every 10 min). Furthermore, the maximum number of messages is also related to energy consumption.

**Figure 2:**
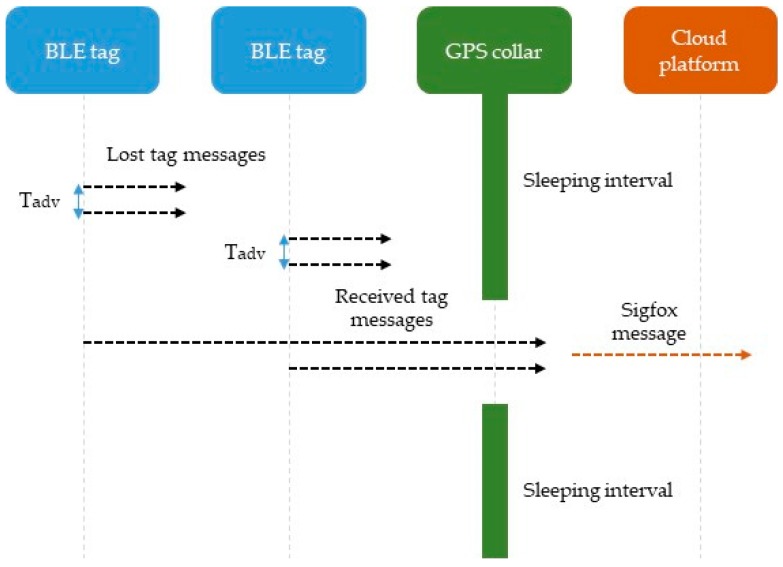
Communication protocol of the proposed solution.

Figure 2 summarizes the communication protocol of the proposed solution. The diagram shows two BLE tags, a GPS collar and the cloud solution. The BLE tags located in the animals send the advertisement messages asynchronously and at regular intervals of several seconds (T_adv_). The GPS collars, placed in other animals, are normally in an idle state, but they are activated asynchronously at regular intervals of several minutes (T_mes_). During this operating window, the advertisement messages sent by the BLE tags are received by the collars. To ensure the reception of the advertisement messages, the operation window must last several times T_adv_. Once the advertisement messages have been received by the BLE module in the collar, they are encoded to ensure their transmission through the Sigfox network. Each message sent by the collar includes GPS location data and identifier-power pairs corresponding to the BLE tags that have been detected. Given the limitation in the size of Sigfox messages, a maximum of eight identifier-power pairs of BLE tags can be sent in each message.

**Figure 3:**
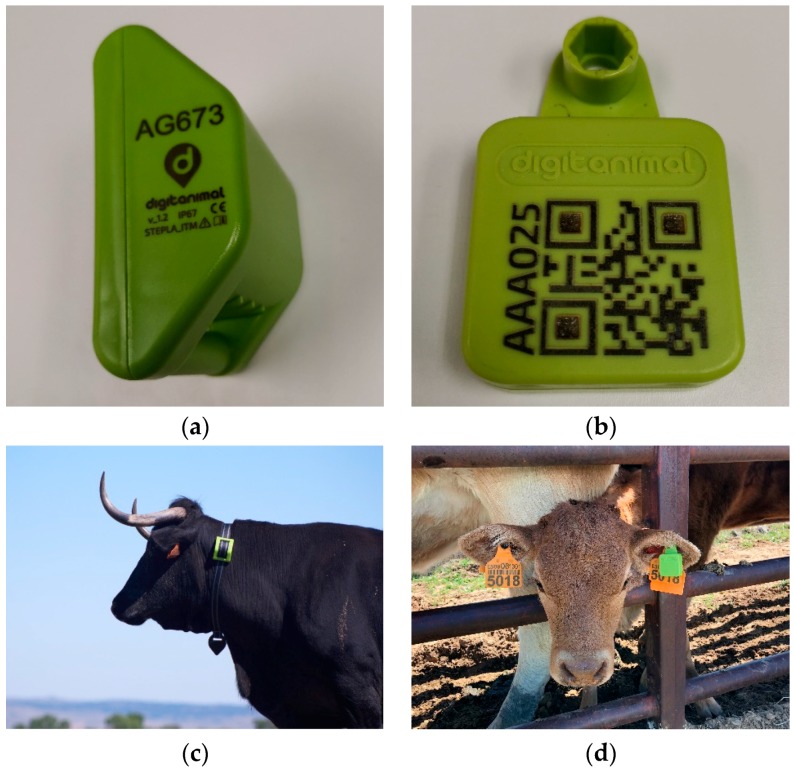
Hardware components of the solution: (**a**) GPS device detail; (**b**) BLE device detail; (**c**) GPS collar placed on the neck of a cow; and (**d**) BLE tag placed on the ear of a calf.

Figure 3 shows the detail of GPS and BLE devices and two examples of animals fitted with them.

### 3.2. System Configuration

Power consumption is a key feature of the proposed system, as it is designed to be used mostly on commercial farms, where animal tracking systems need to provide location data for a long time.

Regarding the BLE tags, there is a trade-off between power consumption and network performance. When the BLE tags broadcast advertisement messages with high power, the coverage is wider, but the operational life is shorter. The same happens with the interval between messages and with the number of repetitions per message. The power consumption of BLE tags in an idle state is 2.0 µA, being 7.5 mA during transmission state, which have been configured to maximize coverage. Considering the battery size that can be used in the ear tag and the need for a long operational life, the BLE tags have been configured with a message interval of one second. This configuration provides an average operational life of 280 days (unpublished data).

As stated, the GPS collars have two operating modes, corresponding to an idle state and an active state. During the idle state, power consumption is less than 20 µA. The power consumption during the active state is due to different components. The GPS unit uses a hot-start mechanism to optimize the consumption, which exceeds 30 mA during satellite search. On the other hand, in order to send Sigfox messages to the server, the system needs 30 mA. Finally, for receiving advertisement messages from the BLE tags, the BLE module have a consumption of 15 mA. Considering all these elements, the collars have been configured to have a temporal resolution of 30 min. The battery life with this configuration exceeds 365 days (unpublished data).

### 3.3. System Validation in Commercial Farms

During the experiment, a total of 70,275 collar messages were gathered from the sheep farm, as well as 78,791 collar messages from the beef cattle farm. This means 28.29 messages per collar per day in the sheep farm and 31.52 messages per collar per day in the beef cattle farm over a total of 48 attempts. As explained before, each of these messages contains data about the location of the collar and the identification of the tags close to it. Altogether, 208,337 tag messages (83.33 reads per tag per day) were gathered from the sheep farm and 93,645 tag messages (37.46 reads per tag per day) from the beef cattle farm.

The KPIs of the system, defined in Section 2.3, are shown in Table 1 as a function of the collar/tag ratio in the herd.

In order to choose a collar/tag ratio, different criteria can be considered, e.g., the number of collars needed to track the location of all tags at least once every day, or the number of collars that is necessary to read tags with a temporal resolution equivalent to that of GPS data.

For both herds, the number of tags read at least once every day increased when the collar/tag ratio was higher. In the sheep farm, 10 GPS collars were enough to detect all the 25 tags on a daily basis. For cows, 25 collars were needed to reach this figure.

Regarding the average time between consecutive readings of the same BLE tag by any GPS collar, this value decreased rapidly as the number of collars increased. In the sheep farm, 15 collars gave an average time between tag readings of 30 min, which is equivalent to the potential temporal resolution of the messages from GPS collars. As 28.29 messages per collar per day where gathered in the sheep farm, the real average temporal resolution was 50.90 min. In the beef cattle farm, the minimum average time between tag readings was obtained with 25 collars. This value (41 min) was very similar to the real average temporal resolution of GPS collars in this farm (45.69 min).

The average number of BLE tags included in each collar message every day is independent of the collar/tag ratio, both for sheep and beef cattle. Nevertheless, this value is higher for sheep, meaning that an ewe has more chances to have other animals at a relatively short distance than a cow.

On the other hand, the proportion of collar messages without BLE tag readings did not change significantly for the different collar/tag ratios (*p* > 0.05). This indicator is lower for sheep, which can be interpreted as ewes being less likely to be separated from the rest of the herd than cows.

Since the sampling scheme of the simulation process was done without replacement, the values for the case of 25 GPS collars correspond to the population mean and, therefore, the resulting standard deviation is zero.

**Figure 4:**
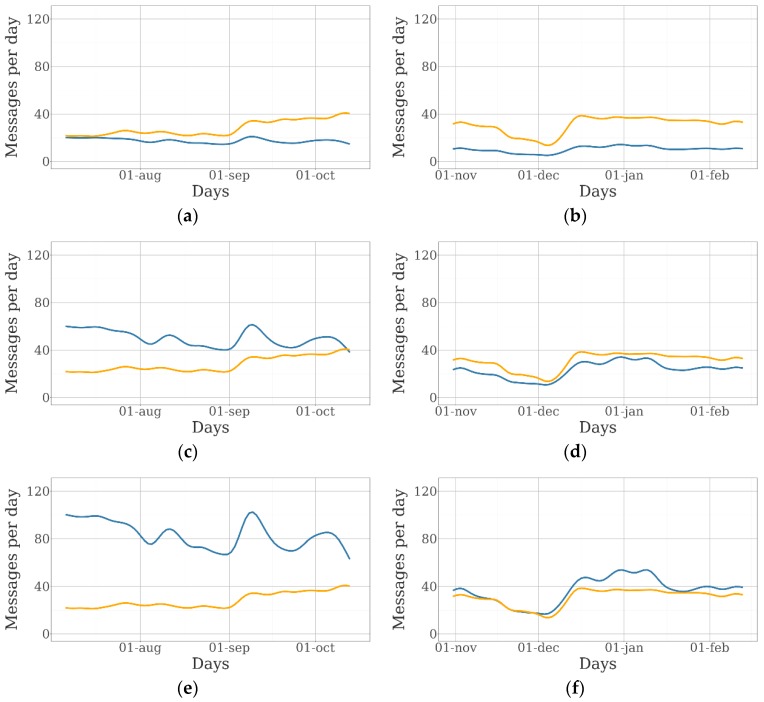
Evolution of the number of messages received daily from GPS collars (orange lines) and BLE tags (blue lines) over the experiment: (**a**) messages from the sheep farm with five collars and 25 tags; (**b**) messages from the beef cattle farm with five collars and 25 tags; (**c**) messages from the sheep farm with 15 collars and 25 tags; (**d**) messages from the beef cattle farm with 15 collars and 25 tags; (**e**) messages from the beef cattle farm with 25 collars and 25 tags; and (**f**) messages from the beef cattle farm with 15 collars and 25 tags.

The charts included in Figure 4 represent the evolution of the number of messages coming from the GPS collars and the BLE tags over the 100-day duration of the experiment for different collar/tag ratios. They have been generated through the random iterative process explained in Section 2.3.

A large variation in the number of messages from GPS collars received per day during the duration of the experiment was observed in both farms. In the sheep farm, this indicator was 23.2 messages per day before September 1st and almost 34.2 messages per day after that date. In the beef cattle farm, the average number of messages per day was 35.2 for the whole period, except for late November and early December, when it was 20.0. The potential number of daily messages was 48, as GPS was activated every 30 min. Animals moved freely around the available grazing area, and the GPS fix-success rate (proportion of scheduled attempts that resulted in a successful location acquisition) as well as the Sigfox fix-success rate (proportion of scheduled messages sent by the Sigfox network), were potentially different in every square meter of the farms. In this case, most of the missing messages were due to the lack of Sigfox coverage, as the GPS fix-success rate was 99.88% in the sheep farm and 97.59% in the beef cattle farm.

**Figure 5:**
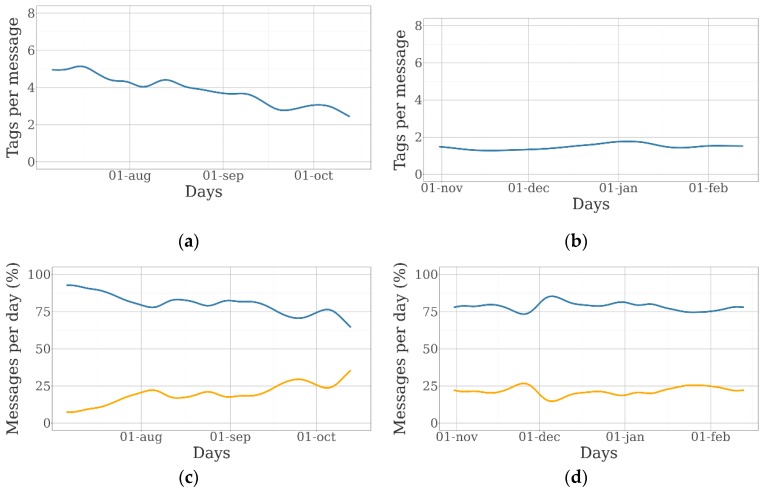
Evolution of the number of tag messages per GPS collar and the percentage of collar messages with (blue line) and without (orange line) over the experiment (25 collars and 25 tags): (**a**,**c**) sheep farm; and (**b**,**d**) beef cattle farm.

The average number of daily messages received from collars remain the same despite the number of collars, which is due to the homogeneity of message-sending rates among devices. Nonetheless, the total number of messages from the BLE tags increased as the number of animals fitted with collars was higher, although the average number of tag messages per collar was the same if the entire experiment is considered. The evolution over the experiment of the number of tag messages per collar and the percentage of collar messages with and without tag information is shown in Figure 5.

The number of tag messages per collar was constant in the beef cattle farm, but a decrease in this indicator was observed in the sheep farm. This effect was due to the displacement of the sheep herd to a different area of the farm, where they grazed in a more dispersed pattern.

**Figure 6:**
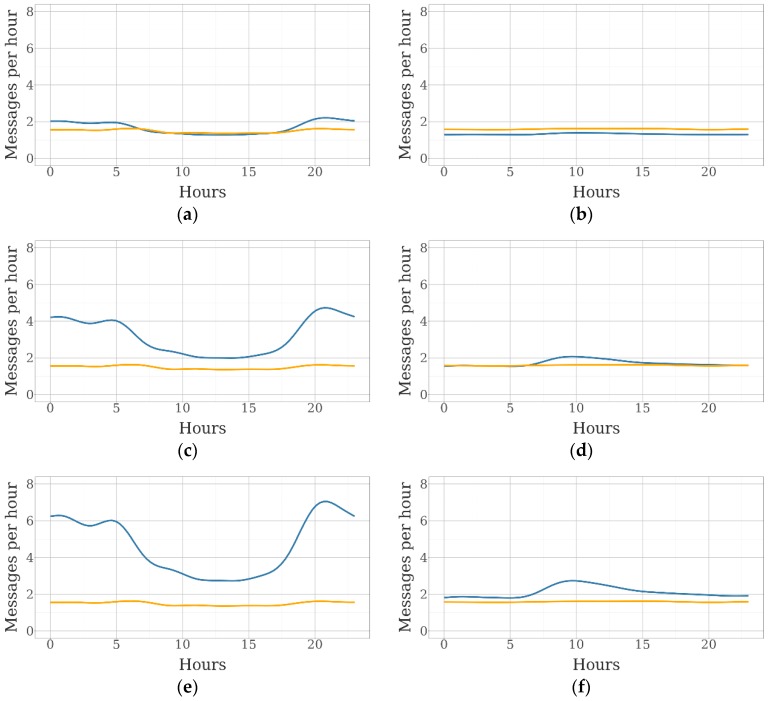
Evolution of the number of messages received hourly from GPS collars (orange lines) and BLE tags (blue lines) over the day: (**a**) messages from the sheep farm with five collars and 25 tags; (**b**) messages from the beef cattle farm with five collars and 25 tags; (**c**) messages from the sheep farm with 15 collars and 25 tags; (**d**) messages from the beef cattle farm with 15 collars and 25 tags; (**e**) messages from the beef cattle farm with 25 collars and 25 tags; and (**f**) messages from the beef cattle farm with 15 collars and 25 tags.

Figure 6 shows the evolution of the number of collar and tag messages within a day.

As mentioned before, the number of tag messages was directly related to the number of collars. In the sheep farm, the number of tag messages was similar to the number of collar messages with five collars. In the case of beef cattle, a greater number of collars was needed for that. A large difference between the number of tag messages received during daylight and at night was observed in the sheep farm. At night, ewes were closer to each other, and the number of tag messages included in each collar message was higher. Therefore, the differences between day and night increased when the number of collars was higher. In the beef cattle farm, the number of messages was quite stable over the day. A peak was observed in the morning, when cows left their rest areas and met each other at the feeding and watering areas.

**Figure 7:**
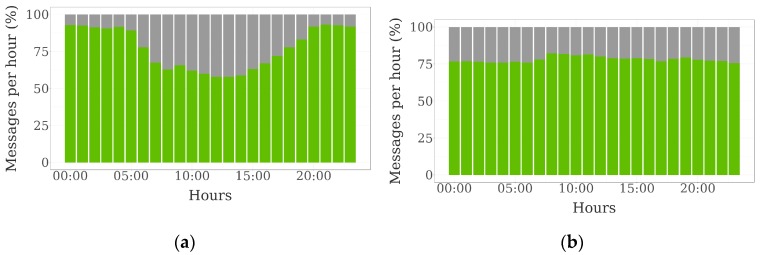
Hourly evolution of the percentage of collar messages with (green) and without (grey) tag data (25 collars and 25 tags): (**a**) sheep farm; and (**b**) beef cattle farm.

Figure 7 represents the evolution of the proportion of collar messages with and without tag data over the day:

In the sheep farm, not only the number of tags included in each collar message was higher during the night, but also the proportion of collar messages without tag data was lower. Few differences over the day were observed in the case of the beef cattle farm.

## 4. Discussion

The system described in the present paper has been designed to be a low-cost and practical solution for commercial farms.

The cost of the GPS collar, which is in the range of 100–150 € per device, is lower than that of similar solutions [32,33,34] and the cost of the BLE tag is at least ten times lower than the cost of the collar. Thus, depending on the collar/tag ratio, the cost per animal can be very low compared to existing solutions. For example, in the case of 10 collars every 25 tags, which was found to be an adequate ratio to have daily data of all sheep in the herd, the cost per animal is 36–54 €. This figure translates into 18–21 € per sheep per year considering a device lifespan of five years, service cost included (battery change and connectivity).

On the other hand, the weight of the GPS-based system (265 g) can be considered low for more than 365 days of operational life, which is longer than that of other low-cost system described in scientific literature [33,35]. In [36] a lighter GPS device to be placed on an ear tag is described, but it has an operational life of a few weeks. In the case of BLE tags, total weight is only 25 g for an average operational life of 280 days. Another important feature for commercial applications is device robustness. The collars were all retrieved with no damage and could be redeployed in other farms. In this regard, several existing solutions place the GPS antenna on the top of the collar [37,38] or into a shoulder-mounted harness [22], because an antenna is most accurate and most efficient when it is pointed towards the sky with nothing obscuring its view [39]. This was considered inadequate for the present solution in terms of robustness and manufacturing cost.

As BLE tags do not provide GPS coordinates, the accuracy of location data gathered from tags is lower than that of collars. Information about the power of the advertisement message is used to compute a radius around the collar position inside which the tag is located. It can be sufficient for most applications in commercial farms and even for some research studies.

The GPS/Sigfox devices used in this work recorded a location fix for 60% (sheep farm) and 66% (beef cattle farm) of all attempts. These results are in the range of those reported by [33] on wild otters using a low-cost GPS/GPRS telemetry system, or by [40] on bison and caribou. Using a store-on-board GPS collar, [38] reported a GPS fix-success rate of 99%, but the terrain was flat and there was no dense tree canopy. Several authors have reported that habitat characteristics, such as topography and tree canopy, may significantly reduce location acquisition [41,42]. Nonetheless, GPS error is a systematic error, which can be computed with many methods or mitigated by some augmented methods, such as ground enhancement signal networks or satellite enhancement networks [43]. In this experiment, although “dehesas” can have a dense tree canopy, most missing location fixes were not due to GPS failures, but to the lack of Sigfox coverage. Despite this, it is important to highlight that signal penetration is better for Sigfox than GSM [44]. On the other hand, regardless of the differences in Sigfox coverage between farms, a tendency has been observed for cow collars to gather more location fixes than sheep collars (unpublished data). It is probably due to the higher position of the GPS antenna when placed on the neck of a cow.

Some authors have previously used short-range communications for tracking animals. Most of the reviewed solutions used passive radio frequency identification (RFID) tags together with antennas placed in strategic points of the building where the animals are housed. Different solutions have been developed and tested, e.g., for laboratory animals [45] or for zoos [46]. The major problem of RFID-based systems is that reading distance is less than a couple of meters, which limits its use to intensive environments. A solution based on active RFID has been tested to track the movements of wild animals (rats) [47], but its need for a network of data loggers distributed throughout the area to be monitored is a major drawback for commercial applications. Bluetooth has also been tested for cattle tracking [48]. Again, the solution described by these researchers is not practical for extensive production systems, since a large number of Bluetooth readers would be needed. The use of animals fitted with collars as network nodes to build mobile ad hoc networks is not new [16]. The ZebraNet project [49] was an early application of opportunistic sensor networks for wildlife monitoring. Wild zebras were fitted with special GPS collars, which sent all its data to encountered neighbors until their own data eventually reached the base station. In the case of the ZebraNet project, the base station consisted of a mobile vehicle for the researchers, which periodically moved around in the savanna and collected the data from the zebras encountered. This solution is quite sophisticated but has two major drawbacks for commercial farm applications. First, the cost is high, as each animal needs to be fitted with a GPS collar. Second, data update needs the vehicle to be moved around. The integration of LPWA networks (LoRa) and opportunistic networks (Bluetooth) has been recently proposed by [50] to overcome the second problem. The solution is similar to that described in the present paper, although these researchers did not test nor validated the system in real-world conditions.

The performance of tag message reception is highly affected by the type of animal due to the differential behavior of sheep and cattle. The gregarious nature of sheep, exhibiting close spatial contact within the flock, has been extensively described in the scientific literature [51]. Beef cows are also gregarious animals, but the distance among animals on the pasture is normally larger than in the case of sheep. Using GPS collars, [35] found that sheep on the same pasture spent 75% of daytime within 51 m from each other, while cattle were within 111 m. These results are coherent with the findings of the present paper, as sheep would be placed inside the theoretical reading range most of the time, but not cows. Consequently, more tag messages would be captured per collar in a flock of sheep than in a cattle herd, as observed. However, the distance among animals on the grazing area does not only depend on the type of animal. Other factors, such as the habitat characteristics, the grass availability, the season or the time of the day, can affect animal behavior [52] and, subsequently, the performance of the proposed solution. Some authors even found behavioral differences in the average distance among sheep in a herd during the day and at night depending on the breed [53].

Thus, there are many factors to be considered in order to choose an optimal collar/tag ratio, which defines the cost per animal of the tracking solution. However, the most important criterion to make that decision is the use of location data. There are several utilities that would require only a location fix per animal per day, or even every few days, e.g., theft or death detection. This data can be obtained with a very low collar/tag ratio, especially in a sheep herd. Farmer management can also be useful to reduce collar/tag ratio. For instance, if animals are housed at night, a few collars could gather messages from lots of BLE tags. It is important to note that, according to the results of this work, the marginal cost of reading one more tag, in terms of additional collars, is high. This is probably because of the limited number of BLE tags used in the experiment. It could happen that these figures were much lower in a more realistic scenario of dozens of collars together with hundreds of tags, considering that a single collar has been able to read 64% of the tags in the sheep herd and 36% of the tags in the beef cattle herd.

Potentially, the proposed solution could be used beyond location monitoring. As it is based on animal proximity to each other, data gathered by the system could be used to study animal affiliations within a herd [54]. This information can be valuable to optimize the collar/tag ratio, e.g., by identifying group leaders and fitting them with the GPS collars. On the other hand, changes in the social structure can be indicators of heat [55], calving [56] and other reproductive and health events of interest for farmers.

## 5. Conclusions

The solution presented in this paper, based on the integration of LPWA (Sigfox) and short-range (BLE) sensor networks, has been demonstrated to be effective in monitoring the location of each animal in a herd at a much lower cost than existing solutions. The collar/tag ratio, which defines the cost per animal of the solution, should be adapted to each case, mostly depending on the utilities associated to animal location data. Further research is needed to develop new utilities for the data gathered by the proposed solution.

## Figures and Tables

**Table 1:** Performance of BLE tag messages reception depending on the number of GPS collars.

Animal Type	No. GPS Collars	No. BLE Tags	Average ± std. No. Tags Read per Day	Average ± std. Minutes between Tag Readings	Average ± std. No. Tags Read per Message	Average ± std. % Messages without Tag Reading
Sheep	1	25	16 ± 2	477 ± 140	4 ± 1	7 ± 5
Sheep	2	25	20 ± 2	232 ± 53	4 ± 1	7 ± 4
Sheep	3	25	22 ± 1	153 ± 30	4 ± 0	7 ± 3
Sheep	4	25	23 ± 1	114 ± 19	4 ± 0	7 ± 3
Sheep	5	25	23 ± 1	91 ± 13	4 ± 0	7 ± 2
Sheep	10	25	25 ± 0	45 ± 4	4 ± 0	7 ± 1
Sheep	15	25	25 ± 0	30 ± 2	4 ± 0	7 ± 1
Sheep	20	25	25 ± 0	22 ± 1	4 ± 0	7 ± 0
Sheep	25	25	25 ± 0	18 ± 0	4 ± 0	7 ± 0
Beef cattle	1	25	9 ± 3	620 ± 276	2 ± 0	17 ± 23
Beef cattle	2	25	12 ± 2	415 ± 99	2 ± 0	16 ± 15
Beef cattle	3	25	14 ± 1	300 ± 55	2 ± 0	16 ± 12
Beef cattle	4	25	16 ± 1	234 ± 37	2 ± 0	16 ± 10
Beef cattle	5	25	17 ± 1	192 ± 27	2 ± 0	16 ± 9
Beef cattle	10	25	21 ± 1	101 ± 9	2 ± 0	16 ± 5
Beef cattle	15	25	23 ± 1	68 ± 4	2 ± 0	16 ± 4
Beef cattle	20	25	24 ± 0	51 ± 2	2 ± 0	16 ± 2
Beef cattle	25	25	25 ± 0	41 ± 0	2 ± 0	15 ± 0

## References

[1] Gordon I.J. (2001). Foreword. Tracking Animals with GPS: An International Conference Held at the Macaulay Land Use Research Institute.

[2] Liu T., Rodríguez L.F., Green A.R., Shike D.W., Segers J.R., Maia G.D.N., Norris H.D. Assessment of cattle impacts on soil characteristics in integrated crop-livestock systems. Proceedings of the American Society of Agricultural and Biological Engineers Annual International Meeting.

[3] Turner L.W., Udal M.C., Larson B.T., Shearer S.A. (2000). Monitoring cattle behavior and pasture use with GPS and GIS. Can. J. Anim. Sci..

[4] Veissier I., Boissy A., Nowak R., Orgeur P., Poindron P. (1998). Ontogeny of social awareness in domestic herbivores. Appl. Anim. Behav. Sci..

[5] Senneke S.L., MacNeil M.D., Van Vleck L.D. (2004). Effects of sire misidentification on estimates of genetic parameters for birth and weaning weights in Hereford cattle. J. Anim. Sci..

[6] Rodgers A.R., Rempel R.S., Abraham K.F. (1996). A GPS-based telemetry system. Wildlife Soc. B.

[7] D’Eon R.G., Serrouya R., Smith G., Kochanny C. (2000). GPS radiotelemetry error and bias in mountainous terrain. Wildlife Soc. B.

[8] Frair J.L., Fieberg J., Hebblewhite M., Cagnacci F., De Cesare N., Pedrotti L. (2010). Resolving issues of imprecise and habitat-biased locations in ecological analyses using GPS telemetry data. Philos. Trans. Roy. Soc. B.

[9] Frair J.L., Nielsen S.E., Merrill E.H., Lele S.R., Boyce M., Munro R.H.M., Stenhouse G.B., Beyer H.L. (2004). Removing GPS collar bias in habitat selection studies. J. Appl. Ecol..

[10] Van Beest F.M., Loe L.E., Mysterud A., Milner J.M. (2010). Comparative space use and habitat selection of moose around feeding stations. J. Wildl. Manag..

[11] Schieltz J.M., Okanga S., Allan B.F., Rubenstein D.I. (2017). GPS tracking cattle as a monitoring tool for conservation and management. Afr. J. Range For. Sci..

[12] Fogarty E.S., Swain D.L., Cronin G., Trotter M. (2018). Autonomous on-animal sensors in sheep research: A systematic review. Comput. Electron. Agric..

[13] Rodgers A.R. (2001). Tracking animals with GPS: The first 10 years. Tracking Animals with GPS: An International Conference Held at the Macaulay Land Use Research Institute.

[14] Evans J.C., Dall S.R.X., Bolton M., Owen E., Votier S.C. (2016). Social foraging European shags: GPS tracking reveals birds from neighbouring colonies have shared foraging grounds. J. Ornithol..

[15] Tomkiewicz S.M., Fuller M.R., Kie J.G., Bates K.K. (2010). Global positioning system and associated technologies in animal behaviour and ecological research. Philos. Trans. Roy. Soc. B.

[16] Pelusi L., Passarella A., Conti (2006). Opportunistic networking: Data forwarding in disconnected mobile ad hoc networks. IEEE Commun. Mag..

[17] Mekki K., Bajic E., Chaxel F., Meyer F. (2019). A comparative study of LPWAN technologies for large-scale IoT deployment. ICT Express.

[18] Raza U., Kulkarni P., Sooriyabandara M. (2017). Low Power Wide Area networks: An overview. IEEE Commun. Surv. Tut..

[19] Berckmans D. (2017). General introduction to precision livestock farming. Anim. Front..

[20] Dieng O., Diop B., Thiare O., Pham C. A study on IoT solutions for preventing cattle rustling in an African context. Proceedings of the Second International Conference on Internet of Things, Data and Cloud Computing.

[21] Nóbrega L., Tavares A., Cardoso A., Gonçalves P. Animal monitoring based on IoT technologies. Proceedings of the IoT Vertical and Topical Summit for Agriculture.

[22] Davis J.D., Darr M.J., Xin H., Harmon J.D., Russell J.R. (2011). Development of a GPS herd activity and well-being kit (GPS HAWK) to monitor cattle behavior and the effect of sample interval on travel distance. Appl. Eng. Agric..

[23] Davis J.D. (2007). Remote Characterization of Locomotion, Grazing and Drinking Behavior in Beef Cattle Using GPS and Ruminant Temperature Dynamics. Ph.D. Thesis.

[24] Moritz M., Galehouse Z., Hao Q., Garabed R. (2012). Can one animal represent an entire herd? Modeling pastoral mobility using GPS/GIS Technology. Hum. Ecol..

[25] Swain D.L., Friend M.A., Bishop-Hurley G.J., Handcock R.N., Wark T. (2011). Tracking livestock using global positioning systems: Are we still lost?. Anim. Prod. Sci..

[26] Liu T., Green A.R., Rodríguez L.F., Ramírez B.C., Shike D.W. (2015). Effects of number of animals monitored on representations of cattle group movement characteristics and spatial occupancy. PLoS ONE.

[27] Castell J.M., Mena Y., Delgado-Pertíñez M., Camúñez J., Basulto J., Caravaca F., Guzmán-Guerrero J.L., Alcalde M.J. (2003). Characterization of semi-extensive goat production systems in southern Spain. Small Rumin. Res..

[28] Moreno G., Pulido F.J. (2009). The functioning, management and persistence of dehesas. Agroforestry in Europe: Current Status and Future Trends.

[29] Adams D.C., Anthony C.D. (1996). Using randomization techniques to analyse behavioural data. Anim. Behav..

[30] Hastie T.J. (2017). Generalized additive models. Statistical Models in S..

[31] R Core Team (2014). R: A language and environment for statistical computing. R Foundation for Statistical Computing.

[32] Zucco C.A., Mourão G. (2009). Low-Cost Global Positioning System Harness for Pampas Deer. J. Wildl. Manag..

[33] Quaglietta L., Herlander-Martins B., de Jongh A., Mira M., Boitani L. (2012). Low-Cost GPS GSM/GPRS Telemetry System: Performance in Stationary Field Tests and Preliminary Data on Wild Otters (Lutra lutra). PLoS ONE.

[34] Knight C.W., Bailey D.W., Faulkner D. (2018). Low-Cost Global Positioning System Tracking Collars for Use on Cattle. Rangel. Ecol. Manag..

[35] McGranahan D.A., Geaumont B., Spiess J.W. (2018). Assessment of livestock GPS collar based on an open source datalogger informs best practices for logging intensity. Ecol. Evol..

[36] Schleppe J.B., Lachapelle G., Booker C.W., Pittman T. (2010). Challenges in the design of a GNSS ear tag for feedlot cattle. Comput. Electron. Agric..

[37] Allan B.M., Arnould J.P.Y., Martin J.K., Ritchie E.G. (2013). A cost-effective and informative method of GPS tracking wildlife. Wildl. Res..

[38] Trotter M.G., Lamb D.W., Hinch G.N., Guppy C.N. (2010). Global navigation satellite system livestock tracking: System development and data interpretation. Anim. Prod. Sci..

[39] Belant J.L. (2009). Effects of antenna orientation and vegetation on global positioning system telemetry collar performance. Northeast. Nat..

[40] Jung T., Hegel T.M., Bentzen T.W., Egli K., Jessup L., Kienzler M., Kuba K., Kukka P.M., Russell K., Suitor M.P., Tatsumi K. (2018). Accuracy and performance of low-feature GPS collars deployed on bison (Bison bison) and caribou (Rangifer tarandus). Wildl. Biol..

[41] Rempel R.S., Rodgers A.R., Abraham K.F. (1995). Performance of a GPS animal location system under boreal forest canopy. J. Wildl. Manag..

[42] Di Orio A.P., Callas R., Schaefer R.J. (2003). Performance of two GPS telemetry collars under different habitat conditions. Wildl. Soc. B..

[43] Lee D., Cho J., Suh Y., Hwang J., Yun H. (2012). A new window-based program for quality control of GPS sensing data. Remote Sens..

[44] Lauridsen M., Nguyen H., Vejlgaard B., Kovacs I.Z., Mogensen P., Sorensen M. Coverage Comparison of GPRS, NB-IoT, LoRa, and SigFox in a 7800 km^2^ Area. Proceedings of the IEEE 85th Vehicular Technology Conference.

[45] Catarinucci L., Colella R., Mainetti L., Patrono L., Pieretti S., Sergi I., Tarricone L. (2014). Smart RFID Antenna System for Indoor Tracking and Behavior Analysis of Small Animals in Colony Cages. IEEE Sens. J..

[46] Kim S.H., Kim D.H., Park H.D. Animal Situation Tracking Service Using RFID, GPS, and Sensors. Proceedings of the Second International Conference on Computer and Network Technology.

[47] Krull C.R., McMillan L.F., Fewster R.M., van der Ree R., Pech R., Dennis T., Stanley M.C. (2018). Testing the feasibility of wireless sensor networks and the use of radio signal strength indicator to track the movements of wild animals. Wildl. Res..

[48] Hartová V., Hart J. (2017). Livestock monitoring system using Bluetooth technology. Agron. Res..

[49] Juang P., Oki H., Wang Y., Martonosi M., Peh L.S., Rubenstein D. Energy-efficient computing for wildlife tracking: Design tradeoffs and early experiences with ZebraNet. Proceedings of the 10th International Conference on Architectural Support for Programming Languages and Operating Systems.

[50] Ayele E.D., Meratnia N., Havinga P.J.M. Towards a New Opportunistic IoT Network Architecture for Wildlife Monitoring System. Proceedings of the 9th IFIP International Conference on New Technologies, Mobility and Security (NTMS).

[51] Lynch J., Hinch G., Adams D. (1992). The Behavior of Sheep: Biological Principles and Implications for Production.

[52] Schoenbaum I., Kigel J., Ungar E.D., Dolev A., Henkin Z. (2017). Spatial and temporal activity of cattle grazing in Mediterranean oak woodland. Appl. Anim. Behav. Sci..

[53] Hewson R., Wilson C.J. (1979). Home Range and Movements of Scottish Blackface Sheep in Lochaber, North-West Scotland. J. Appl. Ecol..

[54] Swain D.L., Bishop-Hurley G.J. (2007). Using contact logging devices to explore animal affiliations: Quantifying cow-calf interactions. Appl. Anim. Behav. Sci..

[55] Corbet N.J., Patison K.P., Menzies D.J., Swain D.L. (2018). Using temporal associations to determine postpartum oestrus in tropical beef cows. Anim. Prod. Sci..

[56] Proudfoot K.L., Jensen M.B., Weary D.M., von Keyserlingk M.A.G. (2014). Dairy cows seek isolation at calving and when ill. J. Dairy Sci..

